# Characteristics of stroke service implementation in Armenia

**DOI:** 10.3389/fneur.2022.1021628

**Published:** 2023-01-12

**Authors:** Greta Sahakyan, Mira Orduyan, Sevak Badalyan, Ani Adamyan, Mariam Hovhannisyan, Hasmik Manucharyan, Sagatel Egoyan, Yuri Makaryan, Hovhannes Manvelyan

**Affiliations:** Department of Neurology, Astghik Medical Center, Yerevan State Medical University, Yerevan, Armenia

**Keywords:** stroke service, Armenia, quality indicator, thrombolysis (tPA), performance measure

## Abstract

**Background:**

Acute stroke care service in Armenia was established in 2019 after the implementation of the National Stroke Program (NSP). This study aimed to provide an up-to-date account of the current image and clinical characteristics of acute stroke service implementation at a tertiary hospital in Armenia by analyzing the quality of care and identifying the areas that need improvement.

**Methods:**

We analyzed patient data from a single hospital in 1 year after the establishment of acute stroke care service (February 2021–January 2022). We selected patients who were within 0–24 h from symptom onset at admission and included patients who benefited from reperfusion therapies (intravenous thrombolysis (IVT) and/or endovascular thrombectomy (EVT)). A favorable outcome was defined as a drop in the National Institutes of Health Stroke Scale (NIHSS) by more than four points at discharge and a modified Rankin score (mRS) of 0–2 at 90 days.

**Results:**

Of the total 385 patients, 155 underwent reperfusion therapies, 91% of patients (141/155) arrived by ambulance, 79.2% (122/155) had neurological improvement at discharge, and 60.6% (94/155) had an mRS of 0–2 at 3 months. Less than 5% of patients had early direct access to the rehabilitation center.

**Conclusion:**

Our study demonstrated that the implementation of NSP with organized protocol-driven inpatient care led to significant advancement in acute stroke service performance. We believe that our report will serve as a model for achieving advanced and structured stroke care in a resource-limited context and contribute to the future development of the healthcare system in our country.

## 1. Introduction

Stroke is the leading cause of acquired physical disability in adults worldwide and the second leading cause of mortality in middle- to high-income countries (1). Moreover, it is a major and challenging healthcare problem, with over 12.2 million new cases each year and over 143 million years of healthy life lost annually due to stroke-related death and disability (2). Current strategies to reduce mortality and disability caused by stroke are based on organized stroke unit care with the implementation of evidence-based clinical guidelines. Patients with stroke who receive organized inpatient care in a dedicated stroke unit are more likely to be alive, independent, and living without disability 1 year after the stroke (3). However, stroke systems of care and the availability of resources for acute stroke care vary considerably across geographic regions, which leads to uneven levels of and, at times, suboptimal care (4).

In a low-resource setting, many barriers to access to appropriate stroke care services are identified, such as inadequate awareness of stroke signs, lack of state-funded medical transportation, non-availability of brain imaging, stroke units and rehabilitation services, low access to health insurance, insufficient training of specialists, and lack of community support in post-discharge care. As a result, morbidity and mortality rates of acute stroke are higher in low- and middle-income countries (LMICs), contributing to 75% of the total death burden (5).

Despite these challenges, there are many efficient models of stroke care available in LMICs, the most frequent ones of which are multidisciplinary team care led by a stroke neurologist, specialist-led care by the general neurologist, physician-led care, stroke telemedicine, and task sharing involving community health workers (6).

Armenia is a country with limited financial resources, resulting in disparities in healthcare coverage among the population. The epidemiology of stroke in the country is neither well-investigated nor controlled, and most of the available data on stroke burden is based on hospital-admitted cases. Though access to the emergency ambulance and primary care service is available to the whole population, there still exists a huge proportion of undiagnosed and non-treated stroke cases like in other developing countries. The reasons for these cases include both, the lack of awareness of stroke symptoms and the seriousness of the situation and possible complications, as well as limited access to urgent medical transportation, inappropriate management and treatment practices by specialists, heterogeneous structure of stroke care services, high cost or non-availability of neuroimaging, and the lack of targeted public and medical staff education (7–9).

Armenia has a population of ~3 million, of which 1.092 million live in Yerevan. (10) Acute stroke care service in Armenia was established in 2019 after the implementation of the National Stroke Program (NSP). The program, funded by the Ministry of Health, is regulated by the experts of the Armenian Stroke Council (ASC) and addresses the hyper-acute and acute management of ischemic stroke. This makes time-sensitive stroke treatment modalities [intravenous thrombolysis (IVT) and/or endovascular thrombectomy (EVT)] accessible to patients.

The Armenian Stroke Council was founded the same year as a collaborative international scientific advisory and educational organization to update the existing stroke service model and to develop accreditation guidelines and procedures for future stroke centers. Members of ASC are experts in the subject of stroke and work in different parts of the world (11). Armenian protocols of stroke unit certification and acute stroke care were formed by the ASC by adapting international guidelines and the American Heart Association/American Stroke Association (AHA/ASA) recommendations to the local needs and characteristics (12). The basic model of stroke care service was based on the reorganization of an existing hospital infrastructure by training health professionals to implement protocol-driven care and multidisciplinary approaches.

Currently, there are four-stroke centers in Armenia, three of which are located in the capital. Thus, there is a huge disparity in access to evidence-based stroke care between capital and rural areas. Moreover, NSP does not cover acute stroke cases with contraindications to reperfusion therapies or cases of hemorrhagic or subacute ischemic stroke. This means that these patients have to pay partially out of their pocket for their inpatient service (“co-payment method”). For this group of patients, there is neither routine control of protocol-based disease management nor regular monitoring of the quality of care.

This study aimed to provide an up-to-date account of the current image and clinical characteristics of acute stroke service implementation at a tertiary hospital in Armenia by analyzing the quality of care and identifying the areas needing improvement.

## 2. Material and methods

A single hospital-based retrospective study design was used to analyze the medical records of patients with ischemic stroke admitted within 0–24 h from symptom onset in 1 year after the opening of acute stroke service in our hospital (February 2021–January 2022). According to the national certification criteria defined by the Ministry of Health, our center is considered a comprehensive stroke center, offering advanced neurosurgical and endovascular procedures for different cerebrovascular pathologies and stroke-related complications. Designed by German architects, the structure of our center is considered to be the most accurate, taking into account the routes between the departments and the rapid transfer system for the patient. We have advanced neuroimaging capabilities available 24 h/7 days, including magnetic resonance imaging (MRI)/angiography 1.5 and 3T, computed tomography (CT)/angiography/perfusion, and conventional digital subtraction angiography. Before 2021, all patients with stroke or other cerebrovascular pathologies were treated in the intensive care unit or general neurology/neurosurgery ward, depending on the clinical state and hemodynamic stability. Dedicated stroke unit care staffed by a multidisciplinary team, including a trained neurologist onsite 24 h/7 days and neuro-intervention staff on call, was established in 2021 after the implementation of NSP. As mentioned earlier, the program addresses time-specific treatment modalities of ischemic stroke, enabling evidence-based protocol-driven care for reperfusion therapy candidates.

To assess the structure and quality of organized stroke unit care, we used the Joint Commission standardized performance measures as a quality indicator in ischemic stroke care (13). We selected patients with hyper-acute or acute ischemic stroke who benefited from reperfusion therapies (IVT and/or EVT). The diagnosis of hyper-acute or acute ischemic stroke was confirmed by neurological examination at admission and neuroimaging results. The mode of imaging (CT angiography/CT perfusion/MRI or all) was selected on an individual basis for every patient by the neurologist in charge. The decision for IVT or EVT was made by following the adapted national guidelines evaluating the risk–benefit ratio for every individual case and after the final agreement of the patient or their family members. Post-procedural care for all patients included non-invasive monitoring and control of vital signs in the stroke unit, assessment of swallowing function by a stroke nurse or speech therapist, assessment of NIHSS score by a neurologist, a follow-up CT at 24 h, etiological workup with a screening of cervical arteries, and echocardiography.

We collected data including baseline demographic and clinical characteristics, patient arrival method (the proportion of pre-notified cases in case of ambulance arrival), the modality of neuroimaging and reperfusion therapy, length of hospital stay (LOHS), in-hospital complication and mortality rate, discharge destination, as well as standardized quality measures including documentation of time of last known well time prior to hospital arrival (LKWT), NIHSS score before recanalization therapy and at discharge, door-to-needle (DTN), door-to-imaging (DIT), and door-to skin puncture (DTP) time, Thrombolysis in Cerebral Infarction (TICI) grade, onset-to-treatment time (OTT), rehabilitation and swallowing assessment within 48 h, administration of antithrombotic therapy by hospital day 2, and stroke education provided during the hospital stay or at discharge. Data were collected by stroke team physicians and clinical residents using prospective registration in the hospital computer system.

Clinical outcome evaluation (modified Rankin score (mRS) at 90 days) was assessed by a standardized telephone interview or during outpatient visits by stroke team neurologists. A favorable outcome was defined as a drop of the NIHSS by more than four points at discharge and an mRS of 0–2 at 90 days. The goal set for the door-to-needle time was defined as <60 min for more than 50% of all IVT cases (14). The analyses were performed using IBM-SPSS statistics.

## 3. Results

Between February 2021 and January 2022, 385 patients were presented to the emergency department of the Astghik Medical Center (AMC) with a suspicion of acute stroke in the first 0–24 h of symptom onset. Of 385 patients, 155 corresponded to the selection criteria of NSP and benefited from reperfusion therapies (IVT, IVT+EVT, and EVT). The median age of the patients was 71. The majority arrived by ambulance (91%), with prenotification of the hospital prior to arrival (89.7%). All patients had documentation of LKWT and NIHSS scores before recanalization therapy. The median DTN time was 60 min, and the median DTP time was 105 min. A total of 98 out of 155 patients (63.6%) had a DTN time of <60 min.

In the EVT group, the proportion of patients with TICI grade 2b or higher was 54 out of 67 patients (85.7%). A total of 143 out of 155 patients (92.3%) had antithrombotic therapy by the end of hospital day 2, and 137 out of 155 patients (87.3%) had an assessment for rehabilitation and swallowing. Stroke education to patients and caregivers was provided in 139 cases (89.6%). Only six patients were directly discharged to the rehabilitation center (3.9%). Furthermore, 122 out of 155 patients (79.2%) had neurological improvement at discharge, and 94 out of 155 patients (60.6%) had mRS of 0–2 at 3 months. In four cases (2.6%), data regarding mRS at 3 months were missing.

Table 1 presents the baseline characteristics of patients and the stroke service performance.

**Table 1:** Baseline characteristics of patients and stroke service.

**Study group characteristics**	***n* = 155**	**%**
Age (Median, IQR)	**71 (64–79)**	
Gender	Female *n =* 74 Male *n =* 81	47.7% 52.3%
Arrival method	*by ambulance, n =* 141 *by own transport, n =* 12 *from another clinic, n =* 2	91% 7.7% 1.3%
Stroke provenance city	***Capital Yerevan, n = 119*** ***Other cities/regions, n = 36***	**76.8%** **23.2%**
Presence of comorbidities	***n =*** **139**	**89.7%**
Hypertension	***n =*** **110**	**71%**
Diabetes type 2	***n =*** **24**	**15.5%**
Cardiac diseases^*^	***n =*** **128**	**82.3%**
IVT	*n =* 88	56.8%
IVT+EVT	*n =* 29	18.7%
EVT	*n =* 38	24.5%
Pre-notification of the hospital before arrival	*n =* 139	89.7 %
Non-prenotified cases	*n =* 16	10.3%
Neuroimaging prior to IVT/EVT	MRI *n =* 25 CT angiography *n =* 105 CT perfusion *n =* 5 MRI+CT angiography *n =* 19	16.2% 67.7% 3.2% 12.2%
LOHS (Median, IQR)		*8 (5–11)*
In-hospital mortality rate	*n =* 14	9%
IVT/EVT-related complication rate ^**^ Symptomatic hemorrhagic transformation	*n =* 11 *n =* 7	7.1% 4.5%
Discharge destination	*Discharge: to home, n =* 134 *to the rehabilitation center, n = 6*	87% 3.9%
NIHSS coverage prior to IVT/EVT	*n =* 155	100%
NIHSS admission (Median, IQR)		11 (7–15)
NIHSS discharge (Median, IQR)		3 (2–6)
DTN time, min.(Median, IQR)		60 (50–80)
DTP time, min.(Median, IQR)		105 (80–120)
DIT, min.(Median, IQR)		16 (10–25)
DTN time <60 min	*n =* 98	63.6%
OTT, min. (Median, IQR)		150 (110–200)
Documentation of LKWT prior to hospital arrival	*n =* 155	100%
Post-EVT TICI grade 2b or higher	*n =* 54	85.7%
Antithrombotic therapy by end of hospital day 2	*n =* 143	92.3%
Stroke Education performance	*n =* 139	89.6%
mRS 5–6 at 3 months	*n =* 23	14.8%
mRS 0–2 at 3 months	*n =* 94	60.6%
Neurological improvement at discharge	*n =* 122	79.2 %
Rehabilitation and swallowing were assessed within 48 h	*n =* 137	87.3%

IVT, intravenous thrombolysis; EVT, endovascular thrombectomy; MRI, magnetic resonance imaging; NSP, National Stroke Program; CT, computed tomography; NIHSS, National Institutes of Health Stroke Scale; DTN time, door-to-needle time; DIT, door-to-imaging time; OTT, onset-to-treatment time: mRS, modified Rankin score; TICI grade, Thrombolysis in Cerebral Infarction grade; DTP time, door-to skin puncture time; NECT, non-contrast-enhanced computed tomography; LKWT, last known well time; LOHS, length of hospital stay; JCI, Joint Commission International.

^*^cardiomyopathy, heart failure, ischemic heart disease, cardiac arrhythmias, and valvular heart disease.

^**^symptomatic brain hemorrhage, major extracranial hemorrhage, orolingual angioedema, and post-puncture femoral hematoma.

## 4. Discussion

Our hospital is the only JCI-accredited clinic in Armenia, and to our knowledge, this is the first report of stroke service quality assessment in our country using performance measures based on Joint Commission standards of care.

A performance measure, as defined by the American Agency for Healthcare Research and Quality, is a mechanism for assessing the degree to which a provider competently and safely delivers the appropriate clinical services to the patient within the optimal period (14, 15). Researchers in both advanced and developing countries have attempted to measure the quality of care by quality indicators adapted to the level of local health service capacity (16). Adherence to these quality indicators is associated with the reduction of death and disability after stroke, thus leading to better stroke care (17).

Our results are encouraging and mostly meet international standards in terms of acute reperfusion therapy management. However, the lack of trained personnel, corresponding infrastructure, quality control, and targeted continuous medical education lead to a heterogeneous structure of stroke service resulting in inappropriate management at different stages of care.

As time is critical for improving stroke outcomes, ASC and local health authorities used major efforts to train emergency staff for appropriate prehospital management.

A recent meta-analysis by Chowdhury et al. showed that prehospital and in-hospital stroke workflow optimizations significantly improve reperfusion rates and time metrics related to stroke treatment. Intervention protocols in prehospital care aim to improve emergency medical system (EMS) response to a stroke by using an EMS stroke survey and/or EMS education, to allow better identification of potential reperfusion therapy candidates. In those cases, activation of a prenotification system to a stroke team enables high-priority triage, allocation of resources, and preparation of the in-hospital pathway (18).

Moreover, in-hospital system interventions are associated with significantly reduced mortality and sICH at 90 days (19).

Currently, there is no national standardized stroke recognition and assessment scale for ambulance physicians in Armenia. Stroke educational programs and training were organized in recent years for ambulance physicians and nurses, but none was provided to emergency call handlers.

A system of prenotification of the stroke service before arrival was established by NSP of a direct phone call from the ambulance physician to the stroke neurologist, enabling better triage and direct instructions regarding prehospital management. Though the majority of patients (91%) arrived at the hospital by ambulance and with prenotification before arrival, there is no available report of the proportion of patients taken to non-stroke-ready hospitals during the first 24 h from symptom onset.

All patients had a clear LKWT documented in their medical history. In about half of the cases, this measure was not appropriately determined by ambulance physicians, possibly due to a lack of corresponding training. NIHSS score was performed and documented for all patients who benefited from reperfusion therapies at admission, at 1 h and 24 h after the therapeutic procedure, and at discharge. Patients who did not undergo revascularization procedures had no NIHSS score recorded in their medical history.

NECT or MRI is recommended within 25 min of the patient's arrival at the emergency department to facilitate the timely administration of intravenous thrombolytic therapy. The goal for DTN time should be established within 60 min for more than 50% of stroke cases (20).

To reduce treatment delays and to optimize in-hospital stroke workflow efficiency, stroke centers must prioritize the implementation of multi-level system interventions, such as prenotification by EMS, direct-to-imaging procedures, bedside IVT administration, education and training, and monitoring and feedback (18).

Our data show that 122 patients (78.7 %) had neuroimaging performance in 25 min, and 63.6 % of patients had DTN time within 60 min. None of our patients had direct access to CT or MRI imaging. IVT was performed in the stroke emergency room. Monitoring and analysis of treatment delays were performed if DNT was longer than 60 min. The reasons for delays were mainly associated with the severity of symptoms and non-stable hemodynamic signs. Our study showed that only five patients out of 155 had CT perfusion, thus highlighting the insufficient use of perfusion imaging in stroke care.

The majority of patients (87.3%) had swallowing and rehabilitation assessment within 48 h of the hospital arrival by a speech and physical therapist. Stroke education was provided for 138 patients and their caregivers (89.6%), addressing all of the following: activation of the emergency medical system, need for follow-up after discharge, medications prescribed at discharge, risk factors for stroke, and warning signs and symptoms of a stroke. Educational materials were provided regarding poststroke care and the prevention of complications. Future studies are needed to evaluate the role of early swallowing and rehabilitation assessment, as well as stroke education, on patient outcomes.

The findings from this study indicate that post-hospital care remains underdeveloped for stroke survivors. Less than 5% of patients were directly discharged to the rehabilitation center. No social or community support was provided in the home setting. No report was found regarding post-discharge occupational therapy. Less than 20% of patients had physical or speech therapy in the outpatient setting.

However, patient outcome based on mRS is encouraging: 60.6% of patients had mRS of 0–2 at 3 months. We believe that the improvement of the poststroke care system in the future will increase this percentage. Moreover, future studies are warranted to evaluate the association of the residual functional deficit with the quality of life and activities of daily living.

We definitely need to expand our access to rehabilitation techniques and develop comprehensive facilities (self-rehabilitation and social reintegration strategies) to support our patient's post-discharge care. The other task must be tight cooperation with patients' primary care physicians to establish an effective framework of patient control and secondary prevention. Third, we suggest specialized neuroradiology training to improve the application of perfusion imaging in our hospital.

Overall, to improve curative and rehabilitative services in separate clinics and enhance the stroke care system in the whole country, it is essential to establish a national stroke registry. We believe that, for better results, the national stroke register should be adapted to address all dimensions of high healthcare quality defined by the World Health Organization (effectiveness, efficiency, accessibility, acceptability, equitability, and safety) (21, 22). Today, the need for a national stroke registry is recognized by healthcare authorities, and steps are being undertaken to arrange the implementation in the future.

### 4.1. Limitations of the study

The limitations of our research include the single-center design, lack of a control group, and relatively small sample size. We only addressed patients who benefited from NSP: we did not include patients with acute hemorrhagic or subacute ischemic stroke. No monitoring was performed for patients with acute ischemic stroke who had contraindications to reperfusion therapies. We hope that in near future, NSP will address these groups of patients, enabling evidence-based and protocol-driven care to all stroke survivors.

## 5. Conclusion

Implementation of an evidence-based stroke system of care in a country with a population of approximately 3 million was in acute demand for many years. Our study demonstrated that despite many challenges, the implementation of NSP with organized protocol-driven inpatient care led to significant advancement in acute stroke service performance. Our results are encouraging and mostly meet international standards in terms of reperfusion therapy management. However, many areas in the stroke care system remain underdeveloped and may negatively affect the outcome of the patients. We believe that our report will serve as a model for achieving advanced and structured stroke care in a resource-limited context and contribute to the future development of the healthcare system in our country.

## Data availability statement

The raw data supporting the conclusions of this article will be made available by the authors, without undue reservation.

## Ethics statement

Ethical review and approval was not required for the study on human participants in accordance with the local legislation and institutional requirements. Written informed consent for participation was not required for this study in accordance with the national legislation and the institutional requirements.

## Author contributions

GS wrote the first draft of the manuscript and is responsible for design, structure, and the final version of the manuscript. MO, SB, AA, MH, and HaM did data collection, data entry, and analysis. SE was responsible for statistical analysis. YM provided critical edits. HoM provided guidance in drafting and is responsible for the final version of the manuscript. All authors read and approved the final manuscript.
